# Combination of CT and RT-PCR in the screening or diagnosis of COVID-19

**DOI:** 10.7189/jogh.10.010347

**Published:** 2020-06

**Authors:** Youxin Wang, Haifeng Hou, Wenrui Wang, Wei Wang

**Affiliations:** 1 Beijing Key Laboratory of Clinical Epidemiology, School of Public Health, Capital Medical University, Beijing, China; 2 Inner Mongolia Comprehensive Center for Disease Control and Prevention, Hohhot, Inner Mongolia Autonomous Region, China; 3 School of Public Health, Shandong First Medical University & Shandong Academy of Medical Sciences, Taian, Shandong Province, China; 4 School of Medical and Health Sciences, Edith Cowan University, Perth, Australia

Coronavirus Disease 2019 (COVID-19), emerged in Wuhan, China, in December 2019. As of 10 March 2020, COVID-19 cases have been reported in 114 countries from all Continents except Antarctica, with accumulative 80 932 cases in China and 29 432 in other countries [1]. The transmission potential of COVID-19, determined by reproduction number (R0) of 3.28, is much higher that of severe acute respiratory syndrome (SARS) [2]. Bold measures taken by China have effectively curbed the rapid spread of this new respiratory disease source and changed the dangerous process of rapid spread of the epidemic [3]. The world is not yet ready to organize and implement the measures that have been proved to be efficient and effective by China to block or minimize the spread of new coronavirus [3]. The crude case-fatality rate (CFR) of COVID-19 is reported to be 2.3% in all patients [4], while higher to be 61.5% in critically ill patients [5]. Therefore, early screening and quarantining mild or asymptomatic cases and early diagnosis of sever patients for intensive treatment are urgent to avoid the pandemic of COVID-19.

Reverse Transcription-Polymerase Chain Reaction (RT-PCR) testing was recommended to confirm COVID-19 cases by China medical authority [6], however the total positive rate of RT-PCR for throat swab samples was about 30% to 60% at initial presentation. Chest Computed Tomography (CT), a routine imaging method, has also been applied to diagnose COVID-19 infection [7]. A study on the correlation of chest CT and RT-PCR testing of COVID-19 based on 1014 cases demonstrated that the sensitivity of chest CT imaging for COVID-19 was 97% (580/601), and the specificity was 25% (105/413), with RT-PCR as a diagnosis criterion [8]. As a new emerging infectious disease, there is no gold criteria for the diagnosis of COVID-19. Our current study showed that if chest CT was taken as a reference of diagnosis standard, the sensitivity of RT-PCR testing for COVID-19 was 65% (580/888), and the specificity is 83% (105/126) (**Table 1**). Thus, if both sensitivity and specificity were taken into account simultaneously, neither chest CT nor RT-PCR testing alone is accurate enough for the diagnosis of COVID-19 infection.

**Table 1:** The performance of RT-PCR for COVID-19 infection with chest CT result as reference

CT	RT-PCR		Sensitivity (95% CI)	Specificity (95% CI)
**positive**	**negative**
Positive	580	308	65% (62%-68%)	83% (76%-89%)
Negative	21	105		

CT – computed tomography, RT-PCR – reverse transcriptase polymerase chain reaction, CI – confidence interval

Considering that asymptomatic cases are also of higher transmission potential, sensitivity should be first considered for the screening purpose of COVID-19 infection. When confirmation for intensive treatment, specificity should be first considered to avoid false treatment. Parallel tests and serial test are needed to increase both sensitivity and specificity. Parallel tests perform RT-PCR and CT imaging at the same time and the results are cross-referenced to make the diagnosis [9]. Serial test employs as a secondary screening test which is performed only if the result of initial screening test is positive [9]. For screening purposes, parallel tests, ie, positive in either RT-PCR or chest CT is used to clinically diagnose COVID-19, can improve sensitivity and decrease false negative cases. For therapy purposes serial tests should be used to improve specificity and decrease false positive cases. Consequently, we recommend that parallel tests are used in screening, while series tests should be used for diagnosis confirmation of COVID-19. The proposed strategic approach for screening and diagnosis confirmation of COVID-19 infection might also be of reference significance for other countries or other emerging infectious disease.

**Figure:**
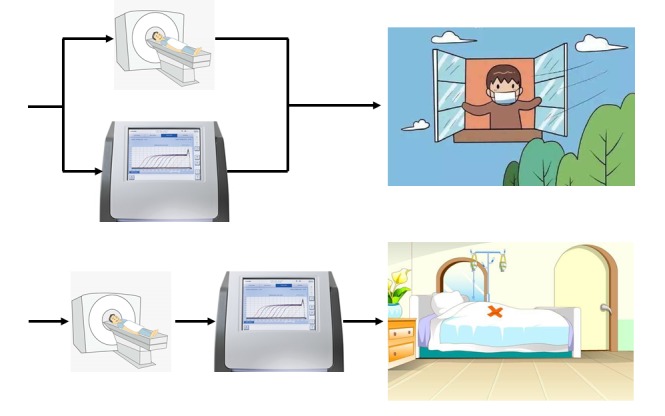
Photo: Parallel or serial test (from the authors’ own collection, used with permission).
